# Simple Matching Using QIIME 2 and RDP Reveals Misidentified Sequences and an Underrepresentation of Fungi in Reference Datasets

**DOI:** 10.3389/fgene.2021.768473

**Published:** 2021-11-26

**Authors:** Lauren E. Eldred, R. Greg Thorn, David Roy Smith

**Affiliations:** Department of Biology, University of Western Ontario, London, ON, Canada

**Keywords:** Basidiomycota, metabarcoding, misidentification, SILVA, sequence identification

## Abstract

Simple nucleotide matching identification methods are not as accurate as once thought at identifying environmental fungal sequences. This is largely because of incorrect naming and the underrepresentation of various fungal groups in reference datasets. Here, we explore these issues by examining an environmental metabarcoding dataset of partial large subunit rRNA sequences of Basidiomycota and basal fungi. We employed the simple matching method using the QIIME 2 classifier and the RDP Classifier in conjunction with the latest releases of the SILVA (138.1, 2020) and RDP (11, 2014) reference datasets and then compared the results with a manual phylogenetic binning approach. Of the 71 query sequences tested, 21 and 42% were misidentified using QIIME 2 and the RDP Classifier, respectively. Of these simple matching misidentifications, more than half resulted from the underrepresentation of various groups of fungi in the SILVA and RDP reference datasets. More comprehensive reference datasets with fewer misidentified sequences will increase the accuracy of simple matching identifications. However, we argue that the phylogenetic binning approach is a better alternative to simple matching since, in addition to better accuracy, it provides evolutionary information about query sequences.

## Introduction

Accurate and reliable identifications of fungi are essential to understanding fungal community structure and ecological functions. However, obtaining precise fungal identifications can be challenging. Fungi can overlap in morphology or can have several different morphs, making it difficult to distinguish between species, even with microscopy (Badotti et al., 2017). Formerly, fungal species had different scientific names for their sexual and asexual morphs. DNA sequencing and phylogenetic analyses have allowed the matching of both morphs and for them to be assigned a single name (Taylor, 2011), enabling easier species identifications without relying on cryptic morphological characteristics (Porras-Alfaro et al., 2014; Lücking et al., 2020). Ecological studies of fungi increasingly involve high-throughput DNA barcoding data, the last step of which is the identification of each sequence, preferably to the species level. This process often relies on specialized identification software with varying degrees of accuracy (Badotti et al., 2017; Lücking et al., 2020).

## Simple Matching and Database Challenges

A major concern when identifying unknown fungi using molecular sequence data is that the reference sequences have been mislabeled, either through misidentification or submission errors (or both). For example, if a user of GenBank’s Basic Local Alignment Search Tool (BLAST) for nucleotides (BLASTn) enters a query sequence that matches to a mislabeled reference sequence, the identification produced would also be incorrect (Kozlov et al., 2016). If the now mislabelled query is then deposited in GenBank, the error is compounded and perpetuated. Unfortunately, reference sequences in GenBank can only be deleted or renamed by the original author(s), making it hard to correct errors (Bidartondo et al., 2008). Consequently, some query sequences may be incorrectly identified with high confidence, or the opposite: assigned a scientific name with low confidence (because of a conflicting match with an incorrect name) when it is actually the correct identification (Lücking et al., 2020). The curation of sequences pertaining to type material by NCBI staff has improved the reliability of identification from BLAST searches (Federhen, 2015; Leray et al., 2019; Macheriotou et al., 2019), but there is still a lot of work to be done.

Simple nucleotide matching *via* the popular bioinformatics software suite “Quantitative Insights Into Microbial Ecology” (QIIME) is particularly sensitive to errors, including misidentifications, when using reference datasets from SILVA or the Ribosomal Database Project (RDP). This is because these datasets contain incorrectly named sequences (Alsammar et al., 2019). The simple matching method involves comparing a query sequence to a group of reference sequences and then choosing the reference sequence with the highest similarity coefficient as the identification (within a specified level of confidence). In studies employing the simple matching method, marker genes (not complete genomes) are used to focus the analysis on short, highly conserved regions (Dalirsefat et al., 2009).

BLASTn is perhaps the most popular simple matching bioinformatics program, but it is impractical and inefficient for analyzing large numbers of environmental sequences, such as those generated by metabarcoding projects (Kim et al., 2005). QIIME 2, an open-source microbiome bioinformatics platform, can perform simple matching of query sequences on very large datasets using its naïve Bayesian Q2 classifier. Moreover, it can employ a variety of molecular reference datasets, including the commonly used SILVA reference dataset. QIIME 2 is an updated and reengineered version of QIIME 1. As compared to its predecessor, it has better visualization features and a clearer demonstration of the inner workings of how the output is generated using a decentralized data-provenance tracking system (Bolyen et al., 2019).

QIIME 2 is commonly used for fungal-marker gene-based analysis (Straub et al., 2020). It can operate in the Virtual Box, which allows users to circumvent the difficulty of installation by providing a functioning QIIME 2 installation inside an Ubuntu Linux virtual machine within Mac or Windows operating systems (Kim et al., 2020). Another simple matching bioinformatics program is the RDP Classifier, which employs the RDP reference dataset, an rRNA dataset of bacteria, Archaea, and fungi. Like the QIIME 2 classifier, the RDP Classifier is a downloadable, open-source package for high-volume query sets. It assigns query sequences to a reference sequence with a bootstrap value estimating the confidence of the assignment. It is fast, requires little memory, does not rely on alignments, and works well with partial sequences. However, because of the highly conserved nature of rRNA genes, the RDP Classifier can only make accurate identifications to the genus level (Cole et al., 2014).

## DNA Barcoding Used in Simple Matching

For the past 3 decades, DNA barcoding (using short sequences of DNA from a coding or noncoding region to identify species) has been instrumental in allowing researchers to accurately identify fungal species (Badotti et al., 2017). There is an important trade-off regarding a marker that is sufficiently conserved to be reliably amplified and aligned but also sufficiently variable to discriminate between closely related species. Marker sequences used in metabarcoding analyses are often limited in size due to the constraints of sequencing technologies, such as those derived from Illumina, Ion Torrent, or 454 sequencing platforms (Heeger et al., 2018). The internal transcribed spacers ITS1 and ITS2 are the most frequently used markers in metabarcoding studies (Lücking et al., 2021). Their usefulness is limited to cases where closely related reference sequences are available, as their phylogenetic signal rapidly reaches saturation. Moreover, for some fungi, including molds and pathogens affecting plants and animals, the ITS regions do not yield sufficient resolution among closely related species (Lücking et al., 2020). Variable regions within the small ribosomal subunit (SSU) rRNA gene, such as the V4 or V6 regions, are also used in metabarcoding, particularly for prokaryotes (Heeger et al., 2018), but they are not sufficiently variable in fungi to differentiate between close relatives. For many fungi, the large ribosomal subunit (LSU) rRNA has more variability and resolution than the SSU and, thus, its 5′ region containing the variable D1 to D3 domains (Heeger et al., 2018) is often used for phylogenetic and metabarcoding (Asemaninejad et al., 2016). Because of sequencing efficiency and nucleotide conservation, these markers are commonly employed to identify environmental sequences of fungi using simple matching.

## Misidentifications by Simple Matching

**FIGURE 1:**
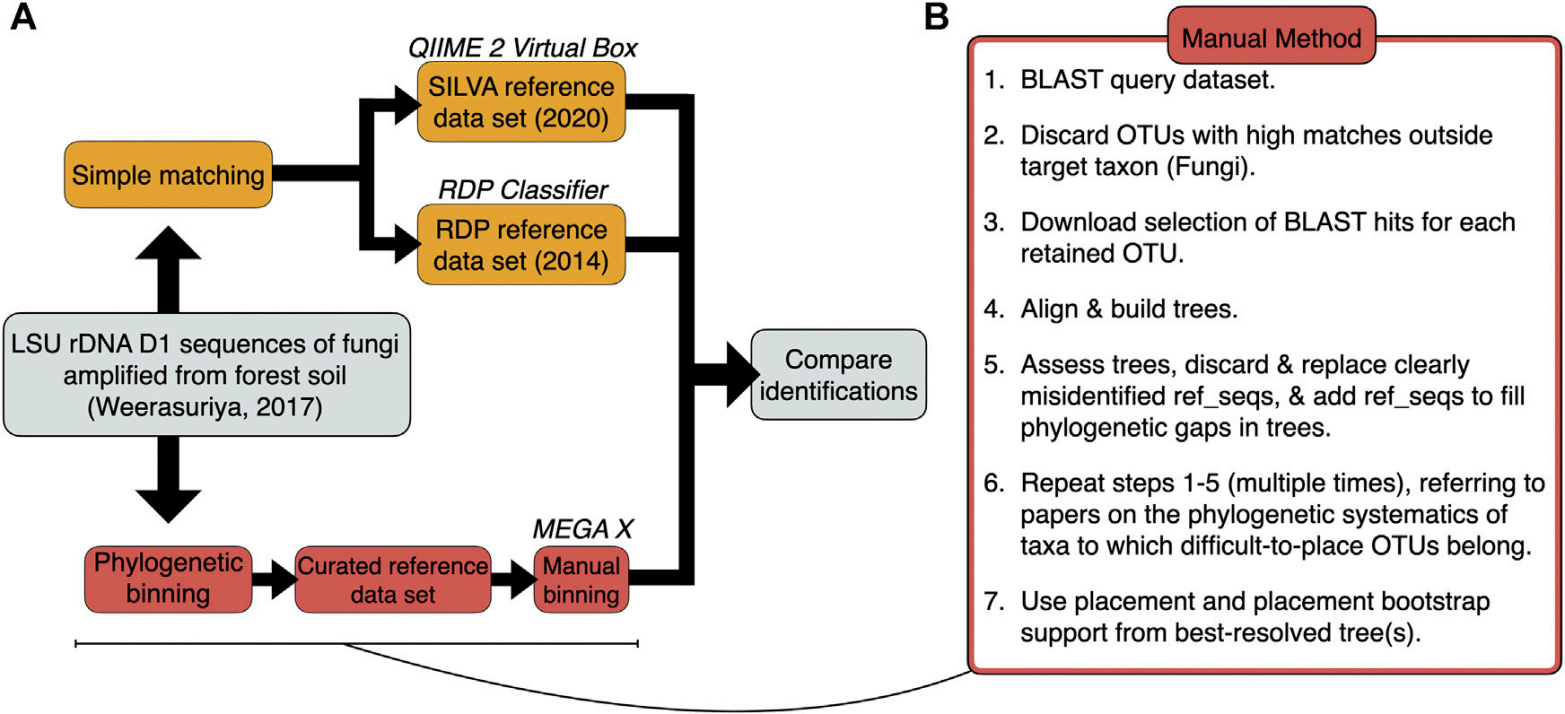
**(A)** Workflow used for the identifications of the 71 query sequences *via* simple matching and phylogenetic binning. **(B)** Step-by-step breakdown of the manual method for phylogenetic binning.

Identifications for 71 query sequences from the Basidiomycota and basal fungi—derived from a representative soil metabarcoding study (Weerasuriya, 2017)—were generated by 1) simple matching using the QIIME 2 classifier and the RDP Classifier with the latest releases of the SILVA (138.1, 2020) and RDP (11, 2014) reference datasets, respectively; and 2) manual phylogenetic binning *via* Multiple Alignment using Fast Fourier Transform (MAFFT) with downstream phylogenetic analyses carried out in the Molecular Evolutionary Genetics Analysis (MEGA X) package (Figures 1A,B; Supplementary Table S1). Manual phylogenetic binning is slower, but it is often a more accurate alternative to simple matching whereby the query and reference sequences are aligned and used to construct a tree with bootstrap values, following which query sequences are placed into “bins” alongside reference sequences (Dröge and McHardy, 2012). Phylogenetic binning can identify fungal species in a sample more effectively than simple matching as it has an additional verification step where the researcher curates the reference sequence dataset from correctly identified, closely related reference sequences (Berger et al., 2011; Matsen et al., 2012; Barbera et al., 2019). In addition to a possible identification, the phylogenetic tree provides a putative framework from which evolutionary information about the query sequences can be deduced (Gregory, 2008).

**FIGURE 2:**
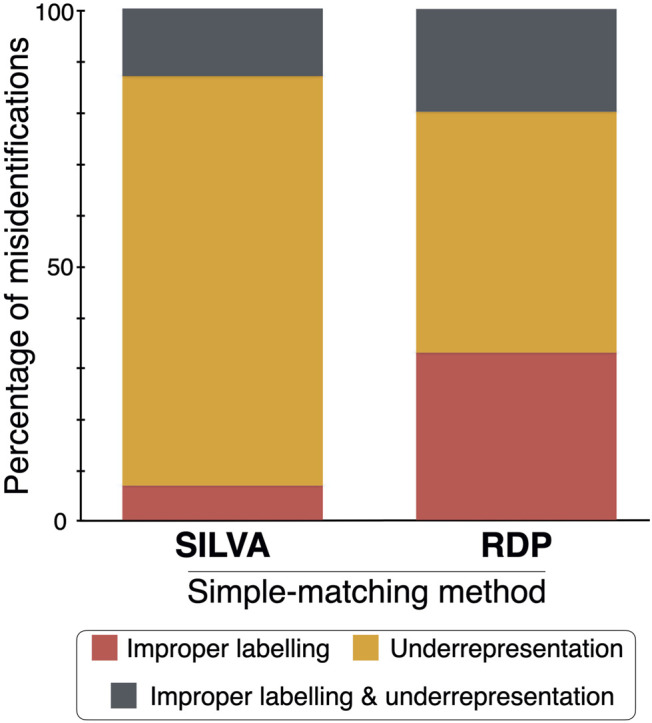
Percentage of the fungal sequence misidentifications in the SILVA and RDP simple-matching methods by either improper labelling, underrepresentation of groups of fungi, or both.

Assuming that the identifications made by manual phylogenetic binning are correct, simple matching *via* the QIIME 2 classifier and the RDP Classifier misidentified 21 and 42% of the 71 operational taxonomic unit (OTU) sequences when the SILVA and RDP reference datasets were used, respectively (Figure 2). The three sequence identification methods (and their associated reference datasets) were then compared to each other by giving an identification score of 5, 4, 3, 2 or 1 for a matching species-level, genus-level, family-level, order-level or class/high-level identification. The sum of these scores for each comparison was divided by 355 (the product of 71 OTU sequences by the highest score of five).

When the two simple matching approaches were compared–the QIIME 2 classifier (using the SILVA reference dataset) and the RDP Classifier (using the RDP reference dataset)–the matching score was 31%. When the QIIME 2 classifier was compared to the manual phylogenetic approach, the matching score was 37.5%. And, finally, when the RDP Classifier was compared to manual phylogenetic binning, the matching score was 42.8%. Hence, the RDP Classifier with its associated RDP reference dataset had more correct identifications than the QIIME 2 classifier when using the SILVA reference dataset.

## OTU-Specific Comparisons

Examination of a few of the OTU-specific comparisons helped to identify the flaws in simple matching (Supplementary Tables S1, S2). For example, OTU 186 was identified as *Coprinopsis strossmayeri* with 100% confidence when using the QIIME 2 classifier with the SILVA reference dataset and as *Coprinopsis cinerea* with 99% bootstrap support when using manual phylogenetic binning. The only *Coprinopsis* species in the SILVA reference dataset is *C. strossmayeri*. The sequence of OTU 186 was queried in GenBank using BLASTn and the top 50 hits were analyzed. In the list of matches, only three were *C. strossmayeri* sequences. When the SILVA reference sequence for *C. strossmayeri* sequence was queried using BLASTn, none of the top 50 matches were *C. strossmayeri.* These findings indicate that the QIIME 2 classifier using the SILVA reference dataset gave a misidentification for OTU 186 due to *Coprinopsis* underrepresentation in the reference dataset and an incorrect identification of the reference sequence. OTU 186 had an identification of *Coprinopsis* with 73% confidence when using the RDP Classifier and the RDP reference set. This genus-level identification is because the dataset contains only sequences identified to the genus level (Cole et al., 2014). Future versions of the RDP reference dataset may eliminate this limitation by adding reference sequences identifiable at the species-level.

Similarly, when using the QIIME 2 classifier with the SILVA reference dataset, OTU 63 was identified as *Cunninghamella bertholletiae* with 93% confidence, whereas when using manual phylogenetic binning it was placed within the *Rhizopus arrhizus* group with 87% bootstrap support. Abe et al. (2010) described the *Rhizopus arrhizus* group as including *Rhizopus oryzae* and *Rhizopus delemar* because of their almost identical LSU D1 regions. When SILVA’s *C. bertholletiae* reference sequence was queried using BLASTn, most of its 50 matches were *Rhizopus* species, without any hits to *Cunninghamella* species. This discrepancy may be the result of an incorrect identification of the *C. bertholletiae* sequence in the SILVA reference dataset, which is truly a *Rhizopus* species. OTU 63 was also correctly identified as *Rhizopus* with 100% confidence when using the RDP reference dataset.

OTU 3 was identified as *Mucor* with 89 and 100% confidence by the QIIME 2 classifier and the RDP Classifier, respectively. Manual phylogenetic binning gave an identification of *Mucor circinelloides* with 80% bootstrap support. All three methods gave a correct identification with high confidence or high bootstrap support. Due to the limitations of their associated reference datasets, the QIIME 2 and RDP classifiers could not give species-level identifications (Cole et al., 2014). With the addition of species-level *Mucor* reference sequences in the SILVA and RDP datasets, the QIIME 2 and RDP classifiers would likely be able to produce an identification similar to that of phylogenetic binning.

OTU 116 was given three different identifications by the three methods: *Phellinus* with 95% confidence (QIIME 2), *Lagarobasidium* with 62% confidence (RDP Classifier) and *Xylodon subflaviporus* with 89% bootstrap support (manual phylogenetic binning). Neither the SILVA nor the RDP reference datasets contained sequences from the genus *Xylodon*. Consequently, QIIME 2 selected a close relative, *Phellinus*, with a similar LSU region using the SILVA reference dataset (Riebesehl et al., 2019). The reference sequence identified in the RDP dataset as *Lagarobasidium* was queried using BLASTn. No matches to *Lagarobasidium* were found and it was determined that the true identification of the sequence is a species in the genus *Xylodon*.

OTU 192 had an identification of *Microstroma* with 100 and 46% confidence when using the QIIME 2 classifier and the RDP Classifier, respectively. Manual phylogenetic binning gave an identification of *Tilletiopsis washingtonensis* with 99% bootstrap support. The SILVA and RDP reference datasets lacked representation of the genus *Tilletiopsis.* Both *Microstroma* and *Tilletiopsis* are in the subclass Exobasidiomycetidae (De Beer et al., 2006). As a result, both of the simple matching classifiers generated the closest identification possible.

This study found that some simple matching reference datasets, namely SILVA and RDP, contain misidentified sequences and suffer from an underrepresentation of certain groups of fungi. As of 2020, fungal sequence data existed for ∼45,000 described species, the majority coming from the ITS regions (Schmit and Mueller, 2007). This represents only 30% of described fungal species and between 1 and 6% of the estimated number of fungal species (Hawksworth and Lücking, 2017). Future mycological work should focus on adding more correctly named, vouchered reference sequences to allow for more comprehensive reference datasets and, thus, more accurate identifications of unknowns. Such an initiative could have significant impacts. For example, correct identification of a clinical isolate of the fungus *Rhizopus arrhizus* is potentially of life-and-death importance, since this species is the main causal agent of COVID-19-associated mucormycosis in India, whereas *Cunninghamella* is more common as a causal agent in Spain (Rudramurthy et al., 2021).

## Updated SILVA and RDP Reference Datasets as Effective Sequence Identification Tools

By analyzing a small set of environmental sequences of Basidiomycota and basal fungi, we conclude that simple matching using QIIME 2 and the RDP Classifier in conjunction with the SILVA and RDP reference datasets is not as effective as manual phylogenetic binning to obtain accurate species-level fungal sequence identifications. This problem occurred because reference datasets contained misidentified reference sequences as well as an underrepresentation of certain groups of fungi and species-level sequences. Improving these reference datasets by eliminating misidentified sequences and adding correctly named representative sequences of a greater fungal diversity would make simple matching a more effective sequence identification tool. Manual phylogenetic binning is currently a more accurate alternative method, although more time consuming, and it also provides additional evolutionary information about query sequence relationships to assist in developing identifications.
