# Supplementary material for: Simple Matching Using QIIME 2 and RDP Reveals Misidentified Sequences and an Underrepresentation of Fungi in Reference Datasets
Source: Front Genet. 2021 Nov 26;12:768473. doi: 10.3389/fgene.2021.768473 (PMC8662557; doi:10.3389/fgene.2021.768473)
Supplement: Supplementary file 1 [file Table1.DOCX]

**Supplementary Table 1**. List of OTU sequences and their identifications through simple-matching (using the SILVA and RDP reference sets) and through manual phylogenetic binning in a maximum likelihood phylogenetic tree of curated reference sequences. Numbers in parentheses represent the identification confidence (%) in simple-matching and the bootstrap support (%) in manual phylogenetic binning.

| OTU Number | SILVA Simple-Matching | RDP Simple-Matching | Manual Phylogenetic Placement |
| --- | --- | --- | --- |
| AOTU 72 | *Mucor* (88) | *Mucor* (100) | *Mucor circinelloides* (80) |
| OTU 3 | *Mucor* (89) | *Mucor* (100) | *Mucor circinelloides* (80) |
| OTU 10 | Mucuromycota (100) | *Thamnidium* (14), Thamnidiaceae (28), Mucorales (100) | *Mucor plumbeus* (98) |
| AOTU 85 | Mucoromycota (100) | *Thamnidium* (14), Thamnidiaceae (32), Mucorales (100) | *Mucor plumbeus* (98) |
| AOTU 176 | *Mucor* (100) | *Thamnidium* (41), Thamnidiaceae (94) | *Mucor* (94) |
| OTU 19 | *Mucor* (100) | *Pilobolus* (46), Pilobolaceae (46), Mucorales (100) | *Mucor mucedo* (88) |
| OTU 32 | *Rhizopus* (100) | *Rhizopus* (100) | *Rhizopus stolonifer* (82) |
| OTU 63 | *Cunninghamella bertholletiae* (93) | *Rhizopus* (100) | *Rhizopus arrhizus* group^1^ (86) |
| OTU 120 | *Lichtheimia* (80) | *Absidia – Lichtheimia* (100) | *Lichtheimia corymbifera* (42)*, Lichtheimia* (100) |
| OTU 5 | *Lichtheimia* (98) | *Absidia* – *Lichtheimia* (100) | *Lichtheimia ramosa* (78) |
| AOTU 88 | *Lichtheimia* (97) | *Absidia – Lichtheimia* (100) | *Lichtheimia ramosa* (78) |
| OTU 0 | *Mortierella* (100) | *Lobosporangium* (61), Mortierellaceae (100) | *Mortierella polycephala* group^2^ (100) |
| AOTU 56 | *Mortierella* (100) | *Lobosporangium* (53), Mortierellaceae (100) | *Mortierella* *polycephala* group^2^ (100) |
| OTU 147 | Basidiobolaceae (81) | *Basidiobolus* (100) | *Basidiobolus microsporus* (24), *Basidiobolus* (100) |

^1^ *Rhizopus oryzae, Rhizopus delemar* (Abe et al. 2010).

| OTU 141 | Chytridiomycota (77) | *Maunachytrium* (55), Lobulomycetaceae (78), Lobulomycetales (78), Chytridiomycetes (89) | Uncultured fungus clone (100), Chytridiomycota (100) |
| --- | --- | --- | --- |
| OTU 184 | Agaricales (100) | *Coprinellus* (50), Psathyrellaceae (95) | *Psathyrella magnispora* (17), *Psathyrella* and *Coprinellus* group (69) |
| OTU 186 | *Coprinopsis strossmayeri* (100) | *Coprinopsis* (73), Psathyrellaceae (84) | *Coprinopsis cinerea* (97) |
| OTU 49 | *Cyathus bulleri* (100) | *Cyathus* (100) | *Cyathus stercoreus* (67), *Cyathus* (99) |
| OTU 104 | Agaricales (100) | *Mycena* (88) | *Mycena leptocephala* (35), *Mycena* (99) |
| OTU 272 | Agaricales (99) | *Cortinarius* (3), Cortinariaceae (17), Agaricales (100) | *Cortinarius cinnamomeus* (45), *Cortinarius* (77) |
| OTU 26 | Agaricales (98) | *Leptonia* (100) | *Entoloma* (99) |
| OTU 81 | Agaricales (89) | *Agaricus* (100) | *Agaricus* (99) |
| OTU 22 | Agaricales (100) | *Lycoperdon* (59), Lycoperdaceae (67), Agaricales (100) | Lycoperdaceae (81) |
| OTU 226 | Basidiomycota (100) | *Clavaria* (86), Clavariaceae (86) | *Clavaria rosea* (89) |
| OTU 95 | Polyporales (79) | *Clavaria* (100) | *Clavaria argillacea. Clavaria sphagnicola* (59*), Clavaria* (99) |
| OTU 55 | Basidiomycota (99) | *Clavulinopsis* (57), Clavariaceae (96) | *Ramariopsis corniculata* (99) |
| OTU 46 | Agaricales (96) | *Hygrocybe* (88) | *Hygrocybe andersonii* (83) |
| OTU 77 | Basidiomycota (100) | Uncultured_Gomphaceae (51), Gomphaceae (94) | *Ramaria rubricarnata* (81) |
| OTU 234 | Basidiomycota (100) | *Mutinus* (90), Phallaceae (100) | *Mutinus elegans* (97) |
| OTU 136 | Basidiomycota (100) | *Ceriporiopsis* (93) | *Irpex lacteus* (99) |

^2^ *Haplosporangium bisporale, Mortierella reticulata, Mortierella polygonia, Mortierella indohii, Mortierella oligospora* (Wagner et al. 2013).

| OTU 115 | Polyporales (100) | *Lentinus* (97) | *Dichomitus squalens* (25), Polyporaceae (71) |
| --- | --- | --- | --- |
| OTU 278 | *Phanerochaete chrysosporium* (100) | *Aphyllophorales* (64), Polyporales incertae sedis (64), Polyporales (79) | *Phanerochaete chrysosporium* (63), *Phanerochaete* (99) |
| OTU 97 | Basidiomycota (100) | Clavulinaceae (77) | *Sistotrema brinkmannii* (99) |
| OTU 150 | Sebacinales (90) | Sebacinales incertae sedis (40), Sebacinales (100) | *Serendipita vermifera* (95) |
| OTU 116 | *Phellinus* (95) | *Lagarobasidium* (62) | *Xylodon subflaviporus* (89) |
| AOTU 93 | Tremellales (100) | *Cryptococcus* (89) | *Vishniacozyma victoriae* (31), *Vishniacozyma* (89) |
| OTU 7 | Tremellales (100) | *Cryptococcus* (92) | *Vishniacozyma victoriae* (31), *Vishniacozyma* (89) |
| OTU 60 | Tremellales (99) | *Kwoniella* (41), Tremellales incertae sedis (41), Tremellales (96) | *Vishniacozyma dimennae* (99) |
| OTU 69 | Tremellales (100) | *Cryptococcus* (70), Tremellaceae (85) | *Saitozyma flava* (89) |
| OTU 44 | Tremellales (99) | *Asterotremella* (100) | *Vanrija humicola* (99) |
| OTU 51 | Tremellales (100) | *Asterotremella* (80), Tremellaceae (80), Tremellales (99) | *Cutaneotrichosporon* (99) |
| OTU 59 | Papillotrema laurentii (100) | *Bullera* (38), Tremellaceae (65), Tremellales (73), Tremellomycetes (73), Basidiomycota (98) | *Trichosporon aquatile* (87) |
| OTU 231 | Tremellales (100) | *Asterotremella* (23), Tremellaceae (23), Tremellales (64), Tremellomycetes (64), Basidiomycota (87) | *Holtermanniella festucosa* (75) |
| OTU 9 | Tremellales (100) | *Asterotremella* (25), Tremellaceae (27), Tremellales (57), Tremellomycetes (57), Basidiomycota (72) | *Holtermanniella takashimae* (83) |
| AOTU 99 | Tremellales (100) | *Asterotremella* (30), Tremellaceae (33), Tremellales (65), Tremellomycetes (65), Basidiomycota (80) | *Holtermanniella takashimae* (83) |
| OTU 50 | Basidiomycota (100) | *Rhodotorula* (11), Microstromataceae (11), Microstromatales (12), Exobasidiomycetes (12), Basidiomycota (96) | *Naganishia adeliensis* (25), *Naganishia* (99) |
| AOTU 108 | Tremellales (100) | *Christiansenia* (41) | *Filobasidium* (93) |
| OTU 11 | Tremellales (100) | *Christiansenia* (42), Christianseniaceae (42), Tremellales (60), Tremellomycetes (70), Basidiomycota (97) | *Filobasidium* (93) |
| AOTU 58 | *Mrakia* (100) | *Mrakia* (100) | *Mrakia gelida, Mrakia blollopis* (83) |
| OTU 111 | *Mrakia* (100) | *Mrakia* (100) | *Mrakia* (83) |
| OTU 4 | *Mrakia* (100) | *Mrakia* (100) | *Mrakia gelida, Mrakia blollopis* (83) |
| OTU 13 | Basidiomycota (100) | *Guehomyces* (100) | *Tausonia pullulans* (97) |
| OTU 17 | Botryobasidiaceae (71) | *Udeniomyces* (100) | *Udeniomyces puniceus* (71) |
| AOTU 66 | *Cystofilobasidium* (100) | *Mrakia* (62), Cystofilobasidiaceae (74), Cystofilobasidiales (74), Tremellomycetes (74), Basidiomycota (100) | *Cystofilobasidium infirmominiatum* (99) |
| OTU 2 | *Cystofilobasidium* (100) | *Mrakia* (63), Cystofilobasidiaceae (75) | *Cystofilobasidium infirmominiatum* (99) |
| OTU 82 | Basidiomycota (85) | *Wallemia* (100) | *Wallemia sebi, Wallemia mellicola* (65), *Wallemia* (99) |
| OTU 21 | Ustilaginomycotina (100) | *Malassezia* (100) | *Malassezia restricta* (99) |
| OTU 35 | Ustilaginomycota (86) | *Malassezia* (100) | *Malassezia globosa* (97) |
| OTU 64 | Ustilaginomycotina (100) | *Malassezia* (100) | *Malassezia pachydermatis* (95) |
| OTU 131 | Basidiomycota (100) | *Malassezia* (99) | *Malassezia japonica* (99) |
| OTU 45 | Ustilaginomycotina (100) | *Malassezia* (99) | *Malassezia* (99) |
| OTU 192 | *Microstroma* (100) | *Microstroma* (46), Microstromataceae (47), Microstromatales (77), Exobasidiomycetes (97) | *Tilletiopsis washingtonensis* (99) |
| OTU 188 | Basidiomycota (79) | *Kurtzmanomyces* (100) | *Kurtzmanomyces* sp. (95) |
| OTU 164 | Basidiomycota (85) | *Kurtzmanomyces* (100) | *Kurtzmanomyces* (99) |
| OTU 90 | Basidiomycota (75) | *Cystobasidium* (80) | *Cystobasidium* (99) |
| AOTU 137 | *Rhodotorula* (100) | *Rhodotorula* (96) | *Rhodotorula* (79) |
| OTU 193 | *Rhodotorula* (99) | *Rhodotorula* (98) | *Rhodotorula* (79) |
| OTU 12 | Microbotryomycetes (100) | *Rhodotorula* (100) | *Rhodotorula mucilaginosa* (99) |
| OTU 34 | Basidiomycota (100) | *Sporidiobolus* (45), Sporidiobolales (45), Microbotryomycetes (100) | *Sporobolomyces* (99) |
| OTU 16 | *Leucosporidium* (100) | *Leucosporidium* (97) | *Leucosporidium fragarium* (12), *Leucosporidium* (97) |
| AOTU 116 | Leucosporidium (100) | *Leucosporidium* (95) | *Leucosporidium fragarium* (12), *Leucosporidium* (97) |
