# Supplementary material for: Simple Matching Using QIIME 2 and RDP Reveals Misidentified Sequences and an Underrepresentation of Fungi in Reference Datasets
Source: Front Genet. 2021 Nov 26;12:768473. doi: 10.3389/fgene.2021.768473 (PMC8662557; doi:10.3389/fgene.2021.768473)
Supplement: Supplementary file 2 [file Table2.DOCX]

| Variable | Database | |
| --- | --- | --- |
|  | RDP | SILVA |
| Presence of Species in Dataset | 14 (33.3%) | 2 (4.8%) |
| Presence of Genus in Dataset | 53 (77.9%) | 26 (38.2%) |
| Identification Success Rate | 0% (55.9%)* | 2.4% (32.4%) |

Supplemental Table 2. Representation of 71 query taxa in reference datasets (RDP 11, 2014) and SILVA 138.1, 2020) and success rate using automated identification with these datasets (RDP in RDP Online Classifier; SILVA using QIIME 2). Of the original 71 query sequences, 42 were identified at the species level and 68 were identified at the genus level by manual phylogenetic binning. These values serve as the sequence comparison baseline for each simple-matching reference set. Note that the RDP classifier does not provide identifications to species.

*From Table S1; first number is success in identifying the query sequence to species; number in parentheses indicates success in identifying the query sequence to genus.
